# Oxygen and mechanical stretch in the developing lung: risk factors for neonatal and pediatric lung disease

**DOI:** 10.3389/fmed.2023.1214108

**Published:** 2023-06-19

**Authors:** Emily Y. Zhang, Colleen M. Bartman, Y. S. Prakash, Christina M. Pabelick, Elizabeth R. Vogel

**Affiliations:** ^1^Department of Anesthesiology and Perioperative Medicine, Mayo Clinic, Rochester, MN, United States; ^2^Department of Physiology and Biomedical Engineering, Mayo Clinic, Rochester, MN, United States

**Keywords:** preterm birth, oxygen, CPAP, mechanical ventilation, asthma, reactive airway disease (RAD), bronchopulmonary dysplasia (BPD)

## Abstract

Chronic airway diseases, such as wheezing and asthma, remain significant sources of morbidity and mortality in the pediatric population. This is especially true for preterm infants who are impacted both by immature pulmonary development as well as disproportionate exposure to perinatal insults that may increase the risk of developing airway disease. Chronic pediatric airway disease is characterized by alterations in airway structure (remodeling) and function (increased airway hyperresponsiveness), similar to adult asthma. One of the most common perinatal risk factors for development of airway disease is respiratory support in the form of supplemental oxygen, mechanical ventilation, and/or CPAP. While clinical practice currently seeks to minimize oxygen exposure to decrease the risk of bronchopulmonary dysplasia (BPD), there is mounting evidence that lower levels of oxygen may carry risk for development of chronic airway, rather than alveolar disease. In addition, stretch exposure due to mechanical ventilation or CPAP may also play a role in development of chronic airway disease. Here, we summarize the current knowledge of the impact of perinatal oxygen and mechanical respiratory support on the development of chronic pediatric lung disease, with particular focus on pediatric airway disease. We further highlight mechanisms that could be explored as potential targets for novel therapies in the pediatric population.

## Introduction

Preterm birth (<37 weeks gestation) remains a significant problem with rates of preterm birth in the US increasing by 0.4% from 10.1% in 2020 to 10.5% in 2021 (1). Thanks to advances in healthcare, the overall mortality for preterm infants has decreased over the past decades and now more than 90% of former premature infants survive into adulthood (2–4). However, prematurity continues to contribute to significant morbidity and chronic health conditions in former preterm infants (4–6). It is therefore increasingly important to investigate and better understand the long-term impacts of premature birth and how early perinatal interventions and insults may contribute to an increased risk of chronic diseases in this vulnerable population.

Chronic respiratory disease remains one of the most significant long-term sequelae of preterm birth. The impact of premature birth on the lung is long-reaching, with increasing evidence that prematurity has a role in development of chronic lung diseases such as asthma and chronic obstructive pulmonary disease (COPD) in adults (7–10). There are numerous innate and extrinsic factors related to prematurity that may impact lung development as well as many perinatal factors such as perinatal infections (chorioamnionitis, postnatal respiratory infections), maternal factors (obesity, diabetes), nutritional deficits, and environmental exposures that may predispose former preterm infants to respiratory disease (11). While all of these factors may play important roles in the development of lung disease, here we will focus specifically on one area of these many factors: the intersection of prematurity and postnatal respiratory support. Preterm infants commonly require respiratory interventions after birth such as oxygen (hyperoxia), mechanical ventilation (MV), or continuous positive airway pressure (CPAP). Unfortunately, these necessary interventions may become contributing factors in development of chronic respiratory disease and its life-long effects.

It is now well-recognized that infants born extremely preterm (<28 weeks gestation) during the late canalicular or early saccular stage of lung development, have the greatest burden of respiratory disease such as bronchopulmonary dysplasia (BPD) (12). However, even children born moderately preterm (32–34 weeks gestation, ~3.6% of births) (13–17) may require interventions such as supplemental oxygen (hyperoxia; typically <60% O_2_) with or without additional mechanical respiratory support which is now most commonly provided in the form of non-invasive nasal CPAP rather than conventional MV (18–25).

While hyperoxia, MV and/or CPAP are necessary for many premature babies (18–27), oxygen and MV are known to contribute to development of BPD (9, 21, 28–33). Antenatal steroids, surfactant, minimizing oxygen exposure, and gentler MV have altered the BPD landscape in recent decades (3, 28, 34–38). However, a major short- and long-term problem for former premies remains chronic bronchial airway disease manifesting as chronic wheezing or asthma (31, 33, 39–47). Here, mild to moderate hyperoxia (<60% O_2_) is most clinically relevant (20, 48–50). And with the now well-established use of nasal CPAP in lieu of conventional MV, it becomes imperative to investigate the intersection of CPAP and supplemental oxygen. Since premature airways are highly compliant and do not undergo additional post-natal development (unlike continued postnatal alveolar development), the effects of CPAP on bronchial airways becomes particularly important. CPAP aids alveolar development and thus BPD may be less of a concern in neonates exposed to CPAP (36, 37, 51–54), but premies without BPD who get oxygen with or without CPAP are still at high risk for airway hyperreactivity (AHR) and associated airway remodeling later in life (45, 55–61).

**Figure 1:**
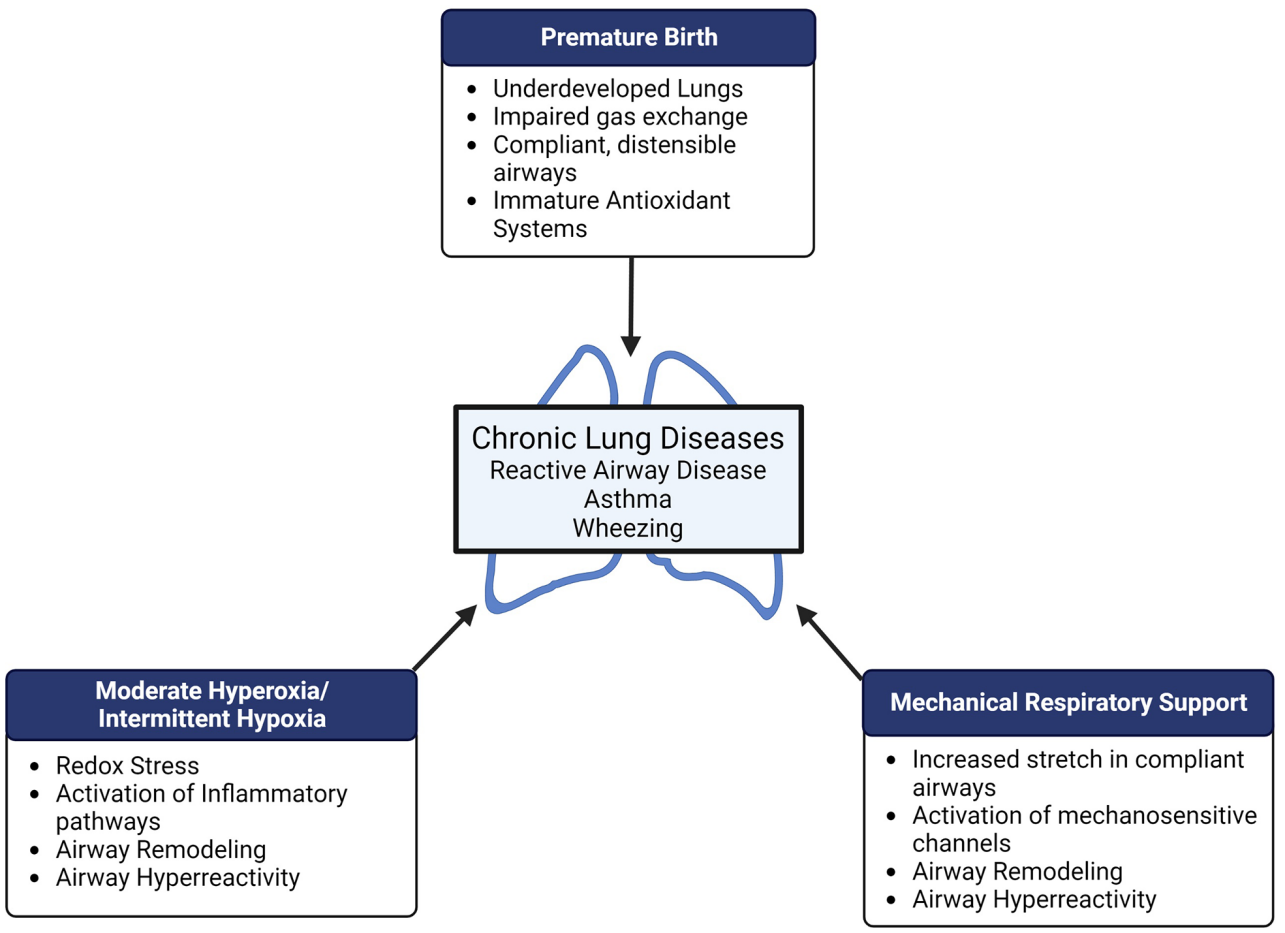
Preterm birth, supplemental oxygen, and mechanical respiratory support all represent potential perinatal “hits” in the developing lung which may result in long-term pulmonary disease. Both hyperoxia and mechanical stretch can cause airway remodeling (increased airway smooth muscle proliferation, increased extracellular matrix deposition/remodeling) and airway hyperreactivity which may contribute to reactive airway disease, wheezing, and asthma in former preterm infants.

In this narrative review, we summarize what is known about the impact of perinatal oxygen and mechanical stretch and the development of neonatal and pediatric airway disease from a mechanistic viewpoint (Figure 1). We also highlight potential future directions for research that may lead to a better understanding of the mechanisms that contribute to development of chronic airway disease, with the hope that new targets may be discovered for novel therapies in the pediatric population.

## Preterm birth and pediatric lung disease

### Lung development during the perinatal period

Chronic lung disease is one of the most common long-term sequelae of preterm birth. Understanding normal perinatal lung development demonstrates why this population has such a high rate of chronic pulmonary disease. Extremely preterm infants [<28 weeks gestational age (GA)] have the highest pulmonary morbidity due to being born during the late canalicular to early saccular stage of fetal lung development (62, 63). During this time, terminal airway branching and formation is completed and the early development of the acinar, gas-exchange regions begins. Type I and II pneumocytes begin to mature and differentiate, distinguishing the surface of alveoli gas-exchange and the surfactant-secreting alveolar cells. Early surfactant formation begins around 24 weeks gestation (62, 63). During the saccular stage (28–36 weeks gestation), the surface area of the lungs starts to expand, laying the foundation for where gas-exchange will eventually take place. Surfactant production does not reach sufficient levels to prevent atelectasis until 32 weeks GA, making very premature infants (28–32 weeks GA) particularly challenged with adaptation to *ex utero* life (62, 63).

During the alveolar stage of lung development (beginning around 36 weeks GA), immature alveoli start to emerge from the sacculi when primary septa elongate into longer and thinner secondary septa. Establishing sacculi into alveoli is the final division of the respiratory tree. Irrespective of whether a baby is born prematurely or at term, the alveolar stage of lung development continues postnatally. Up until approximately 3 years of age, rapid alveolar division and septation is accompanied by an overall increase in lung size (the majority occurring within the first 6 months after birth). This growth, both in alveolar number and size, continues until around 8 years of age. Thus, preterm infants (<36 weeks GA) and very premature infants (<32 weeks GA with insufficient surfactant) have a markedly abrupt interruption in lung development during a period of alveolar septation and multiplicity that now must occur in an *ex utero* environment that these premature infants are ill-equipped to handle (42, 62, 63).

Preterm birth is therefore a significant risk factor for development of chronic lung diseases including BPD, chronic wheezing, and asthma. The risk for developing these lung diseases varies with the degree of prematurity. Extremely preterm infants (<28 weeks GA) are at the highest risk of developing BPD while late preterm infants (33–36 weeks GA) are unlikely to develop BPD but are at increased risk of developing airway diseases such as chronic wheezing and asthma (41, 43, 64, 65).

### Bronchopulmonary dysplasia

Bronchopulmonary dysplasia (BPD) is the respiratory disease most commonly associated with lung diseases of prematurity and has had an evolving pathophysiology and definition as clinical practice has evolved with better understanding of the impact of respiratory support on development of pulmonary disease (66). Due to treatment improvements including incorporation of antenatal steroids, surfactant therapies, less aggressive ventilation, and changes in oxygen exposure, survival of premature infants has increased (67). The incidence of BPD, however, remains the same affecting 10,000–15,000 infants per year in the United States, now predominantly impacting the extremely preterm portion of the population (68).

First reported by Northway et al. (69), the original definition of BPD was based on findings of pulmonary injury, inflammation, and fibrosis due to mechanical ventilation and toxic levels of oxygen supplementation in infants with respiratory distress syndrome. As a result of improved care, younger and very low birth weight infants (<1,500 g) became the predominant population impacted by BPD, with approximately 40% of infants born less than 28 weeks gestation (70) and more than 40% of extremely low birth weight infants affected (71). Severe lung disease now seldomly affects infants >30 weeks GA and > 1,200 g birth weight (72). A “new” BPD definition was developed in 2000 that introduced classifications of none, mild, moderate, or severe BPD based on gestational age and usage of oxygen and other respiratory support (72). This new BPD has a milder clinical manifestation with generally less inflammation, scarring, and damage from ventilation than old BPD (73). New BPD also shows reduced alveolar development that may result from disrupted angiogenesis and alveolar formation in preterm infants born during canalicular or saccular stages when true alveolar formation has yet to occur (73, 74).

### Reactive airway disease

Reactive airway disease, such as wheezing and asthma, is increasingly recognized as an important source of morbidity in former preterm infants. For survivors of BPD, reactive airway disease is a common long-term consequence. Long-term follow-up of extremely premature infants (<26 week GA) diagnosed with BPD showed that about 56% had abnormal spirometry at 11 years of age and 25% were formally diagnosed with asthma (75).

Several studies clearly demonstrate that premature birth is a risk factor for developing life-long chronic diseases of the airway, such as increased airway reactivity, wheezing, and asthma (11, 43, 45, 76, 77). A meta-analysis associated a 30–90% increased incidence of childhood wheezing disorders with preterm birth, with more extreme prematurity aligned with the highest risk of wheezing disorders (45). In one study, children who were born moderately to late premature (32–37 weeks gestational age), continued to have increased wheezing and coughing at 5 years of age (76). Late preterm (33–36 weeks GA) and even term infants with decreased lung function at birth have also been found to have an increased risk of developing reactive airway diseases (78–83).

The pathophysiology of pediatric and neonatal reactive airway disease includes airway remodeling, hyperreactivity, increased airway contractility, and inflammation (11). Airway smooth muscle (ASM) cells and airway epithelial cells are typically most involved in the structural and functional changes of reactive airway disease. Functional changes include loss of airway epithelial barrier function, increased mucous production, and increase in airway smooth muscle cell reactivity. Structural changes involve increased airway smooth muscle cell proliferation and increased extracellular matrix deposition which lead to airway wall thickening and can cause fixed obstruction in addition to the dynamic obstruction caused by ASM hyperreactivity.

## Oxygen and pediatric airway disease

### Perinatal oxygen- transition from *in utero* to *ex utero* life

To understand the impact of oxygen exposure on the preterm lung during the perinatal period, it is important to note the normal physiologic oxygen tension experienced by the fetus during *in utero* lung development. Fetal PaO_2_ (partial pressure of oxygen in the arterial blood) is roughly 25–50 mmHg, whereas maternal PaO_2_ is 80–90 mmHg. The oxygen tension experienced by the fetus changes slightly during various stages of development: embryonic development requires lower PaO_2_ than fetal development later in gestation (84). Physiologic systems monitoring and adjusting for change in *in utero* oxygen tension is critical for normal lung branching morphogenesis, angiogenesis, and extracellular matrix deposition during the pseudoglandular and canalicular stages (85). This tight regulation of oxygen exposure during fetal development is most likely to prevent oxidative stress resulting from too much oxygen exposure during this critical period in development.

During the immediate fetal-to-neonatal transition, there is a sharp increase in fetal PaO_2_ to 70–80 mmHg as the neonate transitions to the 21% oxygen of the *ex utero* environment (86). For full-term healthy infants, arterial oxygen saturation (SpO_2_) typically reaches 95% within 10 min after birth (87). Adjustment to postnatal life comes with challenges in respect to this sudden change in oxygen availability, and there are many factors that can influence this adaptation (e.g., genetics, perinatal inflammation, environmental toxins, maternal diet, and NICU interventions). Importantly, adaptation to postnatal life for a premature infant presents additional challenges due to the underdeveloped nature of the developing lung and immature antioxidant systems.

Because the lungs are underdeveloped in premature infants, the ability to adequately oxygenate and ventilate is seriously impacted. Premature infants in the NICU are frequently administered supplemental oxygen, but the dose and duration of oxygen therapy has been under debate for many years. One reason for this debate is due to the difference in relative oxygen tension experienced by the fetus *in utero* compared to the *ex utero* environment: room air is already a relatively hyperoxic environment for preterm lungs. A second reason oxygen dose and timing has been under debate is due to the known detrimental effects of high O_2_ supplementation on the premature infant (discussed in subsequent sections).

### Hyperoxia and the developing lung

#### Oxygen and BPD

While multiple factors contribute to the pathogenesis of BPD, the role of supplemental oxygen in alveolar simplification and subsequent impaired gas exchange has commanded particular attention. In animal models, hyperoxia exposure alone is sufficient to induce a BPD phenotype (compromised alveolar development and pulmonary vascular remodeling) (88). Animal models of BPD expose neonatal mice to 85–100% O_2_ immediately after birth, resulting in alveolar simplification (89, 90). Additionally, brief exposures of hyperoxia are sufficient to cause long-term structure/function changes in the lung, highlighting the importance of timing O_2_ exposure in accordance with the developmental timeline of the lung such that impact of oxidative stress from a brief O_2_ exposure can be attenuated (91).

Clinically, high levels of oxygen supplementation (80–90%) are now known to be hazardous during prematurity, especially in extreme prematurity, promoting BPD and increasing the likelihood of subsequent interventions. For example, high levels of supplemental oxygen after birth are associated with greater need for ventilatory support (72, 92–95). This concern was initially raised through work done by Saugstad and Vento (92, 96). Studies have shown that newborns receiving even a brief exposure to “supraphysiologic” oxygen during resuscitation have an increased risk of developing BPD, whereas resuscitation with moderate levels of oxygen (30%) resulted in less oxidative stress, inflammation, and incidence of BPD (67, 92). Furthermore, restricting the overall use of supplemental oxygen or implementing lower oxygen saturation targets in preterm infants has been found to result in less inflammation and lower rates of BPD (97).

Growing evidence of the effects of high levels of oxygen during the perinatal period led to modification of supplemental oxygen administration and clinical practice has transitioned to using moderate levels of oxygen in the NICU (30–60% O_2_) (96, 98). However, more recent studies have shown that even moderate levels of oxygen increase the risk for bronchial disease with airway hyperreactivity and remodeling, undoubtedly having long-lasting effects on airway structure and function (22, 43, 45, 99, 100). These changes ultimately lead to the development of asthma and reactive airway disease later in life. We can consider BPD as one of the important risk factors during the perinatal period that contributes to wheezing and chronic airway diseases throughout the lifespan of former preterm infants.

#### Oxygen and reactive airway disease

Due to the established detrimental effects of high concentrations of supplemental oxygen, moderate levels of oxygen are more frequently used in practice. However, even with moderate hyperoxia, there are long-term effects such as increased wheezing and asthma and susceptibility to respiratory infections (22, 43, 45, 99, 100). Chronic airway diseases are characterized by airway remodeling (increased extracellular matrix deposition, ASM proliferation and hypertrophy) and airway smooth muscle hyperreactivity (101). Together, thickened and narrowed hypercontractile airways ultimately cause both fixed and dynamic airway obstruction, leading to the typical clinical symptoms of wheezing and bronchospasm. While studies of BPD have focused primarily on alveoli and the goal of reducing postnatal hyperoxic exposures, there remains a need to better understand the effects of moderate oxygen on bronchial airways, including the impact on airway smooth muscle hyperreactivity and airway remodeling.

There are notable studies demonstrating oxygen-induced effects on airway hyperreactivity that are independent of alveolar injury and show changes that vary with severity (dose and time) of oxygen exposure. For example, neonatal mice exposed to moderate hyperoxia (50%) for 4–7 days immediately after birth followed by 14–16 days of room air ‘recovery’ showed altered lung function (increased airway resistance and decreased airway compliance) during methacholine challenge compared to neonatal mice maintained in room air consistently following birth (42, 100). Importantly, *in vivo* mouse studies comparing 21, 40, and 70% hyperoxia exposures during the first 7 days of life followed by 14 days at room air showed a significant difference in airway hyperreactivity in response to methacholine challenge between the 40 and 70% hyperoxia groups. Neonatal mice exposed to moderate, 40% oxygen from birth to P7 had markedly increased airway resistance (Rrs) and decreased airway compliance (Crs) compared to the control neonates, while neonatal mice exposed to 70% oxygen from birth to P7 exhibited lung function more in-line with the control animals (100). Furthermore, the airways of neonatal mice exposed to 40% oxygen from birth to P7 showed an increased ASM layer compared to both 21 and 70% oxygen exposure groups, implying a particular role for moderate hyperoxia in the pathogenesis of airway remodeling and hyperreactivity (100). This dose dependent variation in ASM proliferation has also been shown in *in vitro* models of hyperoxia exposure, with moderate (40–50%) levels of oxygen resulting in proliferation of developing human ASM cells while high levels (80–90%) of oxygen led to increased apoptosis and cell death (102).

#### Mechanisms of hyperoxic injury

Hyperoxic induced injury in the premature lung is due to ‘oxygen toxicity’ mediated through reactive oxygen species (ROS). Mechanistically, hyperoxic exposures increase ROS and have a myriad of downstream effects including transcriptome changes, insufficient antioxidant defense, mitochondrial overload and dysfunction, and pro-inflammatory responses (103). Each of these processes, detailed below, are interconnected and together contribute to hyperoxia-induced lung injury.

#### Reactive oxygen species

The fetal-to-neonatal transition is naturally a period of physiologic oxidative stress due to the sudden increase in oxygen tension. Imbalanced redox environments pose a challenge for antioxidant systems to regain homeostasis, especially if these systems are underdeveloped such as in the premature infant. Increased levels of ROS become unmanageable and have wide-ranging downstream effects, including mitochondrial dysfunction, changes in gene expression, and inflammatory response. Notably, multiple studies assessing ROS in preterm infants have correlated levels of oxygen exposure with various oxidative stress biomarkers and development of airway disease. Such biomarkers include lipid peroxidation byproducts (92, 104), reduced glutathione (GSH)/oxidative glutathione (GSSG) ratio (92, 105), and protein oxidation ratio of *o*-tyrosine/phenylalanine (92). Furthermore, hyperoxia-driven ROS can lead to altered expression of genes involved in antioxidant systems, inflammation, proliferation, and cell death by regulating transcription factor nuclear translocation, such as HIF, Nrf2, and AP-1 (37, 93, 101, 106–108). ROS can also more directly contribute to oxidative stress by driving oxidation of DNA, proteins, and lipids.

Key factors relevant to the developing lung that are activated in response to oxidative stress include mitogen activated pathway kinases (MAPK), p38, and JNK. Downstream of MAPK signaling is AP-1, a transcription factor involved in regulating cytokine expression, ER stress response, cell proliferation, and apoptosis (107, 109). A recent interest in ferroptosis – iron-mediated programmed cell death – is likely linked to oxidative stress in the developing lung, as downstream effects of ROS include lipid peroxidation. In fact, hyperoxia exposure has been shown to induce ferroptosis in a neonatal mouse model of hyperoxia-induced lung injury (110). *In vivo* studies investigating ferroptosis in hyperoxia-induced lung injury during the perinatal period are relevant to humans. Studies have shown that when mothers receive oxygen supplementation during labor or during elective cesarean section, both the infants and mothers have increased plasma lipid peroxidation byproducts, with neonates having higher arterial and venous umbilical partial pressures of oxygen (86, 111, 112). These emerging studies emphasize the sensitive nature of heightened O_2_ exposure on oxidative stress in the developing fetus.

#### Antioxidant deficiencies and immature defenses

In the mature and healthy lung, antioxidant mechanisms enable adaptation to changes in ROS to regain redox homeostasis under instances of oxidative stress. However, the underdeveloped lung of the premature infant does not have fully mature antioxidant systems. Therefore, premature infants are uniquely susceptible to oxidative stress resulting from hyperoxia-induced lung injury due to deficiencies in antioxidant defenses (67, 113, 114).

The importance of established endogenous antioxidant systems during the neonatal period can be demonstrated in a mouse model of Nrf2 deficiency, in which these mice fared far worse during neonatal hyperoxic lung injury compared to their counterparts with Nrf2 activation (106, 115). In fact, another study showed that adolescents who were born very preterm continue to exhibit markers of oxidative stress (8-isoprostane) in exhaled breath condensate (116), suggesting that effects of prematurity on antioxidant systems last long past the perinatal period.

In a logical therapeutic approach, antioxidant therapies have been tried and tested in the context of prematurity and hyperoxia exposure. Unfortunately, targeting antioxidant systems to decrease ROS overload and oxidative stress in premature infants has been generally unsuccessful. This has been true for studies including either enzymatic or non-enzymatic antioxidant approaches (e.g., targeting N-acetylcysteine as a precursor for *de novo* GSH synthesis) (117–119), targeting superoxide dismutase (SOD) (120, 121), or supplementing antioxidant vitamins such as vitamins A, C, and E (122–125). While these failed attempts at enhancing antioxidant systems in prematurity are perplexing, understanding how these defenses develop during gestation may provide insight. During fetal development, expression of antioxidant enzymes (superoxide dismutase, catalase, glutathione peroxidase) increase dramatically toward the end of gestation (86, 126, 127). A similar pattern is seen for non-enzymatic antioxidant systems: reduced GSH, thioredoxin (TRx), heme-oxygenases, vitamin C, vitamine E, beta carotene, and transition metal chelators all increase in expression and availability toward the end of gestation (86, 126, 127).

The expression and upregulation of these antioxidant systems so close to full-term gestation puts premature infants in a particularly disadvantageous position. It is only days before a fetus reaches full gestation that these enzymatic antioxidants are upregulated and non-enzymatic antioxidants start crossing the placenta (128, 129). In preparation for the transition to a higher oxygen environment, the fetus begins producing its own antioxidants only immediately prior to birth, with another increase in these endogenous systems immediately after postnatal exposure to room air (128–130). The premature infant is therefore hit from three directions: the reliance on maternal antioxidants prior to birth is halted, their ability to preemptively produce antioxidants prior to birth is blunted, and their capacity to induce endogenous antioxidant responses postnatally is inhibited (128, 131).

#### Inflammation

It is important to note that hyperoxia-induced lung injury induces a profound inflammatory response (93, 132–134). This increased inflammatory response is another critical contributor to ROS, airway remodeling, and reduced airway function caused by hyperoxia induced injury (101). Studies have shown that hyperoxia exposure drives pro-inflammatory cytokines and chemokines induced by TLR4 agonists (135). Human studies have shown that bronchoalveolar lavage (BAL) specimens from preterm infants have increased pro-inflammatory mediators (136), and those with BPD also demonstrate increased mast cell and eosinophil levels (101, 137, 138).

As noted previously, perinatal infections have also been implicated in the development of chronic lung diseases. This raises the question of whether synergistic effects may occur between hyperoxia-induced and infection-induced inflammatory cascades. Intriguingly, findings have been mixed and vary with the specific model, timing, and dose of infectious or hyperoxia exposure. In regard to BPD models, multiple animal studies have demonstrated that pulmonary inflammation and alveolar damage are increased with the combination of high levels of oxygen exposure (>85%) and lipopolysaccharide (LPS) or other infectious exposure (95, 139, 140).

The impact of the combination of oxygen and perinatal infectious insults on airway disease has been more mixed. In a mouse model of chorioamnionitis, induced by maternal antenatal lipopolysaccharide (LPS) injection, pups demonstrated increased airway resistance and decreased compliance on pulmonary function testing at 3 weeks of age (132). In the control group not exposed to antenatal LPS, postnatal exposure of pups to 50% O_2_ for the first week of life also resulted in increased airway resistance and decreased compliance. Intriguingly, in a subgroup exposed to both antenatal LPS and postnatal hyperoxia, no synergistic effects were observed (132). Similar results were found in a subsequent mouse study using precision cut lung slices (PCLS) to assess the effects of antenatal LPS and postnatal hyperoxia (141). In this study, both exposures independently increased airway reactivity but there were again no synergistic effects noted.

#### Cell-type effects

Similar to the earlier note regarding effects of hyperoxia being dose-dependent, oxygen effects also vary based on cell type. For example, studies using human fetal airway smooth muscle have shown dose-dependent effects with moderate oxygen increasing proliferation and higher levels (>60%) driving apoptosis (102). Furthermore, studies on human fetal airway smooth muscle have shown moderate hyperoxia increases airway hyperreactivity via intracellular calcium response to bronchoconstrictor agonists (102, 142, 143). *In vivo* studies using moderate hyperoxia in a neonatal mouse model have demonstrated structural and functional changes similar to those seen in asthma and reactive airway disease: increased airway hyperreactivity in response to methacholine challenge as well as increased ASM thickness and collagen deposition in the airway (100, 132). In airway epithelial cells, studies have also shown negative effects of hyperoxia including disruption of the airway epithelial barrier and increased epithelial permeability as well as inflammatory infiltration and fluid accumulation in the lung (135).

### Intermittent hypoxia and the developing lung

While hyperoxia has historically been associated with development of chronic lung diseases of prematurity, an increasing focus has been placed on the role of intermittent hypoxia and hyperoxia in recent years. Intermittent hypoxia occurs commonly in preterm infants due to their immature respiratory control systems that can lead to apnea of prematurity. Apneic episodes in preterm infants typically increase in frequency over the first 2–4 weeks of life, then stabilize and gradually decrease with increasing postnatal age (144–146). During periods of apnea or respiratory pause, hypoxia occurs and may be exacerbated by poor oxygenation due to poor gas exchange in the setting of lung diseases of prematurity (147–149). These hypoxic episodes are typically treated with supplemental oxygen, leading to cycles of hypoxia followed by overshoot hyperoxia. These cycles result in increased oxidative stress and may activate inflammatory cascades, leading to further compromise in alveolar and airway development and increasing risk for development of chronic lung disease.

#### Intermittent hypoxia and BPD

Rodent models of intermittent hypoxia have demonstrated a long-term, deleterious impact on pulmonary function and development of phenotypes similar to those seen in chronic lung diseases of prematurity, particularly mimicking the alveolar simplification seen in BPD. In a preterm rat model of intermittent hypoxia, preterm rats were maintained on 40% oxygen with cycles of 10% oxygen (hypoxia) occurring four times per day (150). Rats exposed to intermittent hypoxia demonstrated decreased maximum expiratory flow rate and decreased lung compliance compared to control or constant 40% hyperoxia exposed mice. Pulmonary epithelial thickening and decreased alveoli and septi branching were noted on histology. HIF-1α and VEGF mRNA and protein levels were found to be increased with intermittent hypoxia (150). An additional study of intermittent hypoxia-hyperoxia in newborn mice found decreased levels of HIF-1α, VEGF, and angiogenic gene expression with intermittent hypoxia-hyperoxia as well as alveolar simplification (151). Finally, a rat model of intermittent hypoxia occurring during recovery from exposure to 60% hyperoxia resulted in decreased alveologenesis as well as pulmonary vascular changes similar to those seen in contemporary BPD (152). Indeed, clinical studies have also found that neonatal intermittent hypoxemia events are associated with development of BPD (153, 154).

#### Intermittent hypoxia and reactive airway disease

Fewer studies have evaluated the impact of intermittent hypoxia-hyperoxia on the developing airway, but initial studies suggest that it may result in persistent changes in airway function similar to those seen in reactive airway diseases. In a rat model of intermittent hypoxia-hyperoxia with preterm rats maintained on 40% oxygen with intermittent hypoxia to 10% oxygen for 7–14 days, maximum expiratory flow rate was decreased compared to room air and straight hyperoxia controls. In another model, neonatal mice were exposed to 10-min repeating cycles of 10% oxygen for 1 min followed by transient exposure to 50% oxygen for 7 days. Following 2 weeks of recovery, the intermittent hypoxia-hyperoxia mice demonstrated increased airway resistance with methacholine challenge as well as decreased baseline compliance compared with normoxic mice (99). Of note, no changes in alveolar or airway structure were found in histologic examination of this model implying that the effects of intermittent hypoxia-hyperoxia may also vary with the dose/duration of exposure.

#### Mechanisms of intermittent hypoxia-hyperoxia

Most studies of intermittent hypoxia-hyperoxia focus particularly on changes in transcription factors termed hypoxia-inducible factors (HIFs, particularly HIF1-α) and vascular endothelial growth factor (VEGF). HIFs are integral to controlling cellular responses to hypoxia or low oxygen tension through modulation of hundreds of downstream pathways involved proliferation, cellular metabolism, angiogenesis, apoptosis, and extracellular matrix formation (155–157). VEGF expression is regulated by HIF1-α. Under hypoxic conditions, VEGF expression increases. Prenatally, VEGF expression is upregulated in the hypoxic *in utero* environment and helps control normal branching morphogenesis and angiogenesis during the pseudoglandular and canalicular stages of fetal development (158, 159). Postnatal disruption of VEGF signaling due to increased oxygen tension has been found to result in decreased alveolar growth and lead to alveolar simplification similar to that found in BPD (160, 161). Lastly, it’s important to consider length of exposure to chronic hypoxia or chronic hyperoxia, and intermittent hypoxia-hyperoxia transitions. *In vitro* studies have shown that there are differential effects of various oxygen exposures on mitochondrial structure and function in developing airway smooth muscle (162). Together, these findings emphasize that the developing lung is rather sensitive to changes in oxygen availability as well as the duration of these exposures.

## Mechanical stretch and the developing lung

In addition to supplemental oxygen, preterm infants commonly require additional respiratory support in the form of mechanical ventilation or CPAP. The mechanical stretch these necessary interventions impose on the compliant and underdeveloped airway and lung may influence lung development and can result in increased risk of both alveolar and airway disease. The lung is an inherently mechanosensitive organ due to the cyclic stretch it undergoes at baseline during normal patterns of breathing. Also, *in utero*, mechanical forces are critical to the normal formation of the developing lung during embryogenesis (163). Peristaltic waves and fetal breathing movements provide the necessary stimuli for normal airway branching and early alveolar development (63, 164–167). It is therefore logical that changes in the mechanical forces the developing lung is exposed to would have bearing on long-term pulmonary health and disease.

Preterm airways have been shown to have greater compliance than the adult and therefore have higher susceptibility to stretch forces and undergo greater distention when exposed to externally imposed strain (168–171). While stretching of the airway is a normal process of fetal breathing and growth, an excessive amount may have damaging effects, leading to inflammatory cascades, alveolar simplification, and even airway hyperresponsiveness (172, 173). Both mechanical ventilation and CPAP impose mechanical stress on the developing lung that may carry implications for development of chronic lung disease.

### Mechanical ventilation

Mechanical ventilation has played a critical role in improving the survival of preterm infants and continues to be an important form of respiratory support, particularly for those born extremely preterm (174, 175). Although a beneficial and life-saving intervention, MV carries risk as well. Inappropriately high tidal volumes or high levels of pressure support can lead to barotrauma or volutrauma and development of ventilator-induced lung injury (VILI) or BPD (176). Even short durations of high-pressure ventilation may result in the initiation of inflammatory cascades and increased cytokine production (173). Alveolar and small airway epithelium development may be disrupted which can lead to acute edema and activation of macrophages (biotrauma) (177). Bronchopulmonary dysplasia development is particularly associated with need for MV. A model with fetal sheep showed lung inflammation characteristic of BPD after only 1 h and alterations in smooth muscle and epithelium at high tidal volume ventilation of 15 mL/kg (173). Furthermore, in a large cohort study of infants weighing less than 1,000 g who were intubated at birth it was found that the severity of BPD increased with the duration of mechanical ventilation (178).

### Continuous positive airway pressure

In light of the known risks associated with conventional MV, current care has shifted toward use of non-invasive interventions such as nasal CPAP when possible (179). CPAP has been associated with reduced need for surfactant administration, decreased need for conventional MV, and decreased risk of BPD and death in preterm infants with respiratory distress syndrome (RDS) (18, 180). When compared to surfactant treatments, need for postnatal corticosteroids or intubation were also lessened with CPAP, while the rate of BPD stayed constant (181, 182). Despite potential improvements compared to conventional MV, CPAP still results in increased pressure and potential distention in the compliant, developing airway and the long-term effects of this mechanical stimulus on the developing airway require further investigation. Initial data in animal models suggest that CPAP is not a completely benign therapy and may also result in long-term changes in airway structure and function consistent with reactive airway disease.

In a mouse model of neonatal CPAP, neonatal mice were exposed to 3-h cycles of CPAP for the first 7 days of life followed by 2 weeks of recovery. Lung slices taken from 8-day old mice immediately after a week of CPAP exposure demonstrated increased airway reactivity in small airways of male, but not female, mice. Both male and female mice demonstrated increased airway hyperreactivity to methacholine challenge after 2 weeks of recovery, implying a persistent impact of neonatal CPAP exposure on the developing airway (183). In a subsequent mouse study, neonatal CPAP exposure was found to increase expression of the extracellular calcium-sensing receptor (CaSR) and inhibition or knockdown of CaSR was found to blunt CPAP effects on airway contractility, implying one potential mechanism for CPAP effects on ASM (172).

The consequences of stretch have also been investigated *in vitro* through examination of the impact of stretch on ASM and fibroblast cells. Multiple studies have demonstrated that mature human ASM cells proliferate and increase migration in response to cyclic mechanical stretch (184–186) and cyclic mechanical stretch of 5–10% has also been shown to increase proliferation of human embryonic lung fibroblasts (187). Intriguingly, in the embryonic fibroblasts, a higher amplitude stretch of 15–20% decreased cell proliferation but increased extracellular matrix (ECM) collagen production implying a potential dose–response (187). Other studies have also found that stretch impacts ECM deposition, remodeling, and composition. Mature human ASM expression and activation of matrix metalloproteases 1, 2, and 3 (MMPs, regulators of ECM remodeling) were found to be increased by mechanical stretch (184). Exposure to 10 or 30% compressive strain increased deposition of collage II and collagen IV in a 3D airway model consisting of bronchial epithelial cells seated on a collagen matrix embedded with fibroblasts (188). In this model, expression and activity of MMPs 2 and 9 were also increased (188). While these *in vitro* models predominantly use cyclic stretch, they demonstrate the impact of changes in mechanical stimulation on remodeling behavior in airway cells and the airway ECM.

### Mechanisms of stretch-induced responses

Multiple pathways have been implicated in stretch-induced remodeling responses in airway smooth muscle. Transforming growth factor-β1 (TGF-β1) has been shown to contribute to both airway smooth muscle hypertrophy and proliferation, potentially contributing to airway remodeling changes prominent in asthma (189, 190). Adult human airway smooth muscle cells exposed to 12% strain for 1–24 h demonstrated increased TGF-β1 mRNA expression (190). This stretch-induced increase was mitigated by blockade of PTK, PI3K, and MEK1/2 mediated pathways. Rho/Rho-kinase pathways have also been implicated in ASM hyperresponsiveness and remodeling, mediated in part through unidentified stretch-sensitive ion channels (190–192). In a neonatal mouse model of CPAP-induced airway hyperreactivity, the extracellular calcium-sensing receptor was found to be a potential mediator of CPAP-stretch induced airway hyperreactivity (172). CPAP exposure of neonatal mice increased CASR expression as well as airway reactivity to bronchoconstrictor agonists while blockade of CASR blunted the CPAP-induced increased in airway reactivity. Finally, interactions between ASM and the ECM have been shown to be important, with particular focus on stretch-induced changes in actin polymerization and cytoskeletal remodeling and communication with the ECM (193, 194).

While numerous pathways have been shown to contribute to stretch-induced remodeling and ASM hyperreactivity, the upstream, stretch-sensitive mechanisms underlying these stretch-induced changes have yet to be well-elucidated. Here, increasing our understanding of some of the mechanosensitive channels present in the lung may provide new possibilities for therapeutic targets. Because of the intrinsic mechanosensitivity of the lung, it contains numerous types of mechanosensitive channels, each with different roles and function. A few key families of channels have begun to emerge as potentially relevant to reactive airway disease and response to stretch. Here, two families of mechanosensitive channels have emerged with particular relevance: transient receptor potential vanilloid-type channels (particularly TRPV4) and piezo (PZ) channels.

#### TRPV4 channels

TRPV4 is a mechanosensitive channel that has been found in airway epithelium and airway smooth muscle and has been shown to regulate embryonic lung development and airway tone (195–198). During lung development, TRPV4 helps regulate branching morphogenesis as well as contractility (197). TRPV4 activation causes Ca^2+^ influx that can activate pathways involved in cell proliferation and migration (199) and is also involved in regulation of smooth muscle contraction (200–202). Given the importance of TRPV4 for *in utero* lung development as well as its role in regulation of Ca^2+^ influx and mature smooth muscle cell contraction and migration, TRPV4 could be a potential regulator of immature, postnatal airway response to mechanical stretch, such as occurs with CPAP.

#### Piezo channels

The second family of mechanosensitive channels that warrant further investigation are piezo (PZ) channels. PZ channels were first described about a decade ago and are non-selective cation channels that are conserved across species and present in multiple organ systems (203–205). Little is known about the role of PZ channels in the lungs but, similar to TRPV4, both PZ1 and PZ2 have been found to play important roles in lung development. PZ1 is critical for development of normal vascular architecture with deletion of PZ1 disrupting pulmonary vascular development and proving lethal in mice (205, 206). PZ1 has also been found to play an important role in triggering surfactant secretion in ATI cells in response to stretch, triggering ATII cells through paracrine stimulation (207). PZ2 has also been found to be important in the developing lung and is crucial for adequate respiratory function and proper lung expansion in neonatal mice (208). Newborn mice with Piezo2 deficiency develop respiratory distress and death without respiratory support, implying an important role for PZ2 in sensing stretch in the immature airway (208). Better understanding of the role PZ channels in the developing lung is needed and could potentially lead to discovery of new therapeutic targets to prevent the long-term impact of mechanical stretch in the development of chronic pediatric pulmonary disease.

### The intersection of oxygen and stretch

There is surprisingly little that is known about the intersection of supplemental oxygen and mechanical respiratory support, even though they are commonly administered in tandem for preterm infants with respiratory distress in the NICU. This is in part due to the independent complexity of the impact of these two therapies. As evidenced by the studies presented previously, both oxygen and stretch have differential impacts on the developing airway that vary greatly with the “dose” and duration of exposure. But the question that logically arises is whether the combination of CPAP and hyperoxia has a synergistic impact on the developing lung. Are their effects additive- or is it possible that one somehow mitigates the impact of the other? One study in neonatal mice found that when CPAP or moderate hyperoxia (40%) were administered alone to neonatal mice (P1-7) they developed increased airway reactivity (209). But when moderate hyperoxia and CPAP were administered together, this increase in airway reactivity no longer occurred. However, airway wall thickness was still increased in all three groups (209). Further investigation is needed to better understand how the combination of oxygen and mechanical stretch intersect in the developing lung and what the long-term ramifications are for preterm infants exposed to these necessary and common therapies.

## Summary and conclusion

Despite numerous advances in care, preterm infants remain at significantly increased risk of developing chronic pulmonary diseases including BPD and reactive airway diseases such as wheezing and asthma. This increased risk stems from the fact that preterm lungs are underdeveloped, with poor baseline gas-exchange due to interrupted alveolar development. They are further ill-equipped to adapt to the redox stress of a hyperoxic *ex utero* environment due to immature antioxidant systems. Finally, they are also disproportionately exposed to additional perinatal insults including supplemental oxygen and mechanical respiratory support that further increase the risk of developing chronic lung disease. While numerous studies have shown the deleterious impact of high levels of oxygen on alveolar development, data continue to emerge on the differential impact of more moderate levels of oxygen exposure, particularly in regard to the risk of developing chronic bronchial airway disease. Similarly, little is known about the impact of CPAP-induced stretch on the developing lung and airway. A better understanding of the impact of these evolving therapies on the developing lung may open new avenues for novel therapies to treat or prevent the development of chronic pediatric lung diseases.

## Author contributions

EV, CP, and YP contributed to conception and design of the manuscript. EV, CP, EZ, and CB wrote sections of the manuscript. All authors contributed to manuscript revision, read, and approved the submitted version.

## Funding

This work was supported by a Foundation for Anesthesia Education and Research Mentored Research Training Grant (EV), NIH grants R01 HL56470 (YP) and R01 HL160570 (CP and YP), and the Department of Anesthesiology and Perioperative Medicine, Mayo Clinic, Rochester.

## Conflict of interest

The authors declare that the research was conducted in the absence of any commercial or financial relationships that could be construed as a potential conflict of interest.

## Publisher’s note

All claims expressed in this article are solely those of the authors and do not necessarily represent those of their affiliated organizations, or those of the publisher, the editors and the reviewers. Any product that may be evaluated in this article, or claim that may be made by its manufacturer, is not guaranteed or endorsed by the publisher.
